# A wavelet features derived radiomics nomogram for prediction of malignant and benign early-stage lung nodules

**DOI:** 10.1038/s41598-021-01470-5

**Published:** 2021-11-16

**Authors:** Rui Jing, Jingtao Wang, Jiangbing Li, Xiaojuan Wang, Baijie Li, Fuzhong Xue, Guangrui Shao, Hao Xue

**Affiliations:** 1 Department of Radiology, Second Hospital of Shandong University, Jinan, Shandong People’s Republic of China; 2 Department of Biostatistics, School of Public Health, Shandong University, Jinan, Shandong People’s Republic of China; 3 Department of Cardiology, Shandong Provincial Hospital, Jinan, Shandong People’s Republic of China; 4 Department of Radiology, Second Hospital of Shandong University Zhaoyuan Branch, Zhaoyuan, Shandong People’s Republic of China; 5 Department of Neurosurgery, Qilu Hospital of Shandong University, Jinan, Shandong People’s Republic of China

**Keywords:** Cancer, Biological techniques, Cancer

## Abstract

This study was to develop a radiomics nomogram mainly using wavelet features for identifying malignant and benign early-stage lung nodules for high-risk screening. A total of 116 patients with early-stage solitary pulmonary nodules (SPNs) (≤ 3 cm) were divided into a training set (N = 70) and a validation set (N = 46). Radiomics features were extracted from plain LDCT images of each patient. A radiomics signature was then constructed with the LASSO with the training set. Combined with independent risk factors, a radiomics nomogram was built with a multivariate logistic regression model. This radiomics signature, consisting of one original and nine wavelet features, achieved favorable predictive efficacy than Mayo Clinic Model. The radiomics nomogram with radiomics signature and age also showed good calibration and discrimination in the training set (AUC 0.9406; 95% CI 0.8831–0.9982) and the validation set (AUC 0.8454; 95% CI 0.7196–0.9712). The decision curve indicated the clinical usefulness of our nomogram. The presented radiomics nomogram shows favorable predictive accuracy for identifying malignant and benign lung nodules in early-stage patients and is much better than the Mayo Clinic Model.

## Introduction

Solitary pulmonary nodules (SPNs) are high-incidence intrapulmonary lesions; an SPN usually refers to a lesion with a diameter ≤ 3 cm that is round with no atelectasis, no satellite lesions, and no local lymphadenectasis^1,2^. Qualitatively diagnosing SPNs as benign or malignant has important clinical significance, could avoid the high risk of surgery for benign SPNs, and could also allow early surgical treatment of malignant SPNs to improve patient survival^3^. At present, CT is simple and economical to apply. Multi-slice spiral computed tomography (MSCT) has high spatial and density resolution, which can improve the sensitivity and specificity of detection of SPNs^4^. The high diagnostic accuracy of MSCT makes this imaging modality suitable to be widely used for diagnosing benign and malignant SPNs^3^.

Radiomics is a high-throughput extraction method for images that depends on large numbers of imaging features and subsequent quantitative analysis. Radiomics shows excellent decision-making capacity for disease diagnosis and prognostic prediction^5^. Currently, radiomics of lung nodules is mainly used to improve the nodule detection rate^6^ or clinical decision-making^7–12^, and enhanced CT can be used^13,14^. To our knowledge, few ideal radiomics-based studies evaluating the prediction of early-stage (≤ stage I) malignant and benign lung nodules ≤ 3 cm has been published to date because of their more indistinguishable radiomics features.

In this study, we used one original feature and nine wavelet radiomics features out of 788 features and validated a novel radiomics nomogram that incorporated a radiomics signature and clinical risk factors to distinguish malignant and benign early-stage SPNs.

## Materials and methods

### Patients

A total of 116 patients (116 SPNs) were enrolled in our study from Jan 2016 to Dec 2018, and the recruit pathway is presented in Fig. S1. and the patients had no anti-tumor therapy before surgery. Their CT images were retrospectively analyzed and found to show SPNs that had confirmed pathological results. A total of 116 SPNs were detected with LDCT imaging. After surgery, the TNM stage of lung cancer was confirmed to be T1N0M0. Patients were divided into training and validation set in a ratio 3:2. 70 patients were divided into training set and 46 patients were divided into validation set.

This retrospective study was approved by the ethics review board of Second Hospital of Shandong University. The requirement for informed consent was waived by our Review Board (Second Hospital of Shandong University) owing to the retrospective nature of the current study. The methods in the current study were performed in accordance with the relevant guidelines and regulations.

### CT image acquisition, region-of-interest segmentation, and radiomics feature extraction

Before undergoing pulmonary nodule resection or biopsy, all patients underwent pulmonary plain CT with a GE 64-slice spiral CT scanner (LightSpeed VCT 64, General Electric Company). The CT scan parameters were as follows: 0.7 s/r of rotation time of the X-ray tube, voltage of 120 kV, current of 100 mA, pitch of 0.2, and collimation of 0.6 mm × 64. The conventional scanning slice thickness was 5 mm, while the reconstructed slice thickness was 1.5 mm. The pulsmonary window had a window width of 1500 HU and a window level of − 600 HU. The mediastinal window had a window width of 350 HU and window level of 35 HU. The images were transmitted to a picture archiving and communication system (PACS) system. Two chest radiologists with more than 10 years of experience in image diagnosis read, analyzed and diagnosed the original thin-layer (1.5 mm) images on the workstation and recorded the chest CT manifestations.

Tumor regions of interest (ROI) were semiautomatically segmented slice by slice using 3D Slicer (www.slicer.org). Two chest radiologists with more than 10 years of experience in image diagnosis read, analyzed and diagnosed the original thin-layer (1.5 mm) images on the workstation and recorded the chest CT manifestations. A large set of quantitative radiomics features were extracted using the PyRadiomics^15^. In total, 788 radiomics features were extracted from a single CT image. 100 radiomics features which were extracted from original image could be divided into three categories: (a) first-order statistics features, (b) shape-based features, (c) statistics based textural features. The remain 688 radiomics features were extracted from images with wavelet transformation, and therefore were called wavelet features. More detailed information about the radiomics features and their extraction reproducibility can be found in the Supplementary Data.

Interclass correlation coefficients (ICCs) were used to assess the intra- and interobserver reproducibility of radiomics feature extraction. An ICC greater than 0.75 indicates good agreement of the feature extraction.

### Feature selection in benign and malignant early-stage SPNs and radiomics signature construction

We used the least absolute shrinkage and selection operator (LASSO) logistic regression algorithm to select early SPN benign and malignant related feature with nonzero coefficients from the 788 imaging features in the training set^16^. A formula was generated using a linear combination of selected features that were weighted by their respective LASSO coefficients; the formula was then used to calculate the radiomics score for each patient to reflect the risk of malignancy. Finally, the predictive accuracy of the radiomics signature was quantified by the area under the receiver–operator characteristic (ROC) curve (AUC) in both the training and validation sets. The association between the selected features and lung nodule malignancy were investigated using univariable logistic regression model.

### Construction and assessment of the radiomics nomogram

The radiomics signature and the clinical variables were tested in a multivariable logistic regression model to identify benign and malignant early-stage SPNs in the training set. A radiomics nomogram was then constructed based on the multivariate logistic regression model. The calibration of the nomogram was assessed with a calibration curve. The Hosmer–Lemeshow test was performed to assess the goodness-of-fit of the nomogram, and the area under the curve (AUC) was calculated to quantify the discrimination performance of the nomogram. In addition, the predictive importance of each variable was assessed using the respective *t* statistics value in the radiomics nomogram.

Internal validation of the radiomics nomogram was performed with the validation set. A radiomics score was calculated for each patient in the validation set using the formula constructed in the training set. Calibration and the Hosmer–Lemeshow test were performed, and the AUC was calculated.

### Clinical utility of the radiomics nomogram

To estimate the clinical utility of the nomogram, decision curve analysis (DCA) was performed by calculating the net benefit for a range of threshold probabilities in the combined training and validation sets.

### Statistical analysis

The LASSO logistic regression model was used with penalty parameter tuning that was conducted by fivefold cross-validation on the training set based on maximal AUC criteria. The likelihood ratio test with backward step-down selection was applied to the multivariable logistic regression model. Detailed descriptions of the LASSO algorithm and DCA are provided in the Supplementary Data.

All statistical tests were performed using R statistical software version 3.5.2. We used the "glmnet" package to perform the LASSO logistic regression model analysis. The ROC curves were plotted using the "pROC" package. The 95% confidence intervals of AUC were estimated using the “ci” function in the "pROC" package. Nomogram construction and calibration plots were performed using the "rms" package, and the Hosmer–Lemeshow test was conducted using the "generalhoslem" package. The predictive importance of variables were calculated using the “varImp” function in the “caret” package. DCA was performed using the " rmda " package. A two-sided *P* < 0.05 was considered significant.

## Results

### Patient clinical characteristics

**Figure 1:**
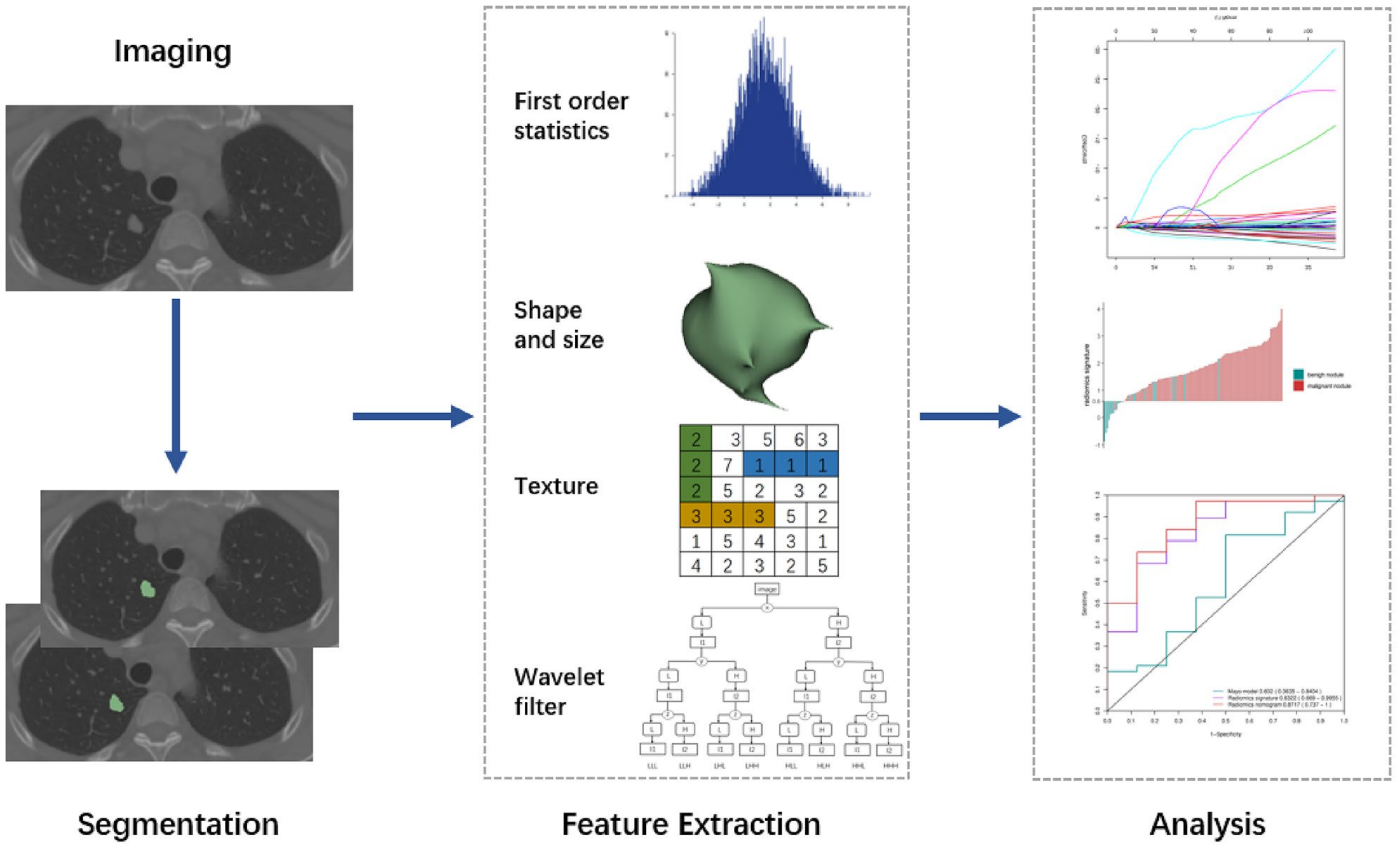
Radiomics study flowchart. Radiomics flowchart. (**A**) Nodules were manually segmented on plain CT images. (**B**) Three categories of radiomics features were extracted from original CT, and wavelet features were extracted after wavelet decomposition. (**C**) After features selection, the most informative radiomics features and clinical features were combined to construct machine learning model. Model performance was assessed using ROC, calibration curve, DCA and et.al.

The radiomics study flowchart is presented in Fig. 1. The patient characteristics in the training and validation sets are shown in Table 1 and Supplementary Table S1. Patients with malignant SPNs accounted for 81.4% (57/70) and 82.6% (38/46) of the training and validation sets, respectively, and there were no significant differences between them. Gender showed no significant differences between the benign and malignant groups, but age showed certain significant differences in our study.

**Table 1:** Baseline characteristics of the training and validation sets.

Characteristic	Training set (*N* = 70)		*P*	Validation set (*N* = 46)		*P*
	Malignant (*N* = 57)	Benign (*N* = 13)		Malignant (*N* = 38)	Benign (*N* = 8)	
Age, mean ± SD, years	62.25 ± 9.83	55.08 ± 9.44	0.017	62.95 ± 8.85	58.63 ± 14.53	0.265
Gender (%)			> 0.999			> 0.999
Male	33 (57.90)	8 (61.54)		17 (44.74)	4 (50.00)	
Female	24 (42.10)	5 (38.46)		21 (55.26)	4 (50.00)	

### Feature selection, radiomics signature construction and performance

A total of 788 imaging features were extracted from each images: 100 features were divided into three categories: 18 first order statistics features, 14 shape and size features and 68 textural features; the 678 features were classified as the fourth category, which contain all first-order statistics features and textural features but were extracted from images with wavelet decomposition. More detailed information about the imaging features can be found in the Supplementary Material S2 and Fig S3. The quartile of interobserver ICCs is [0.8866, 0.9431], indicating favorable intra- and interobserver reproducibility of feature extraction.

**Figure 2:**
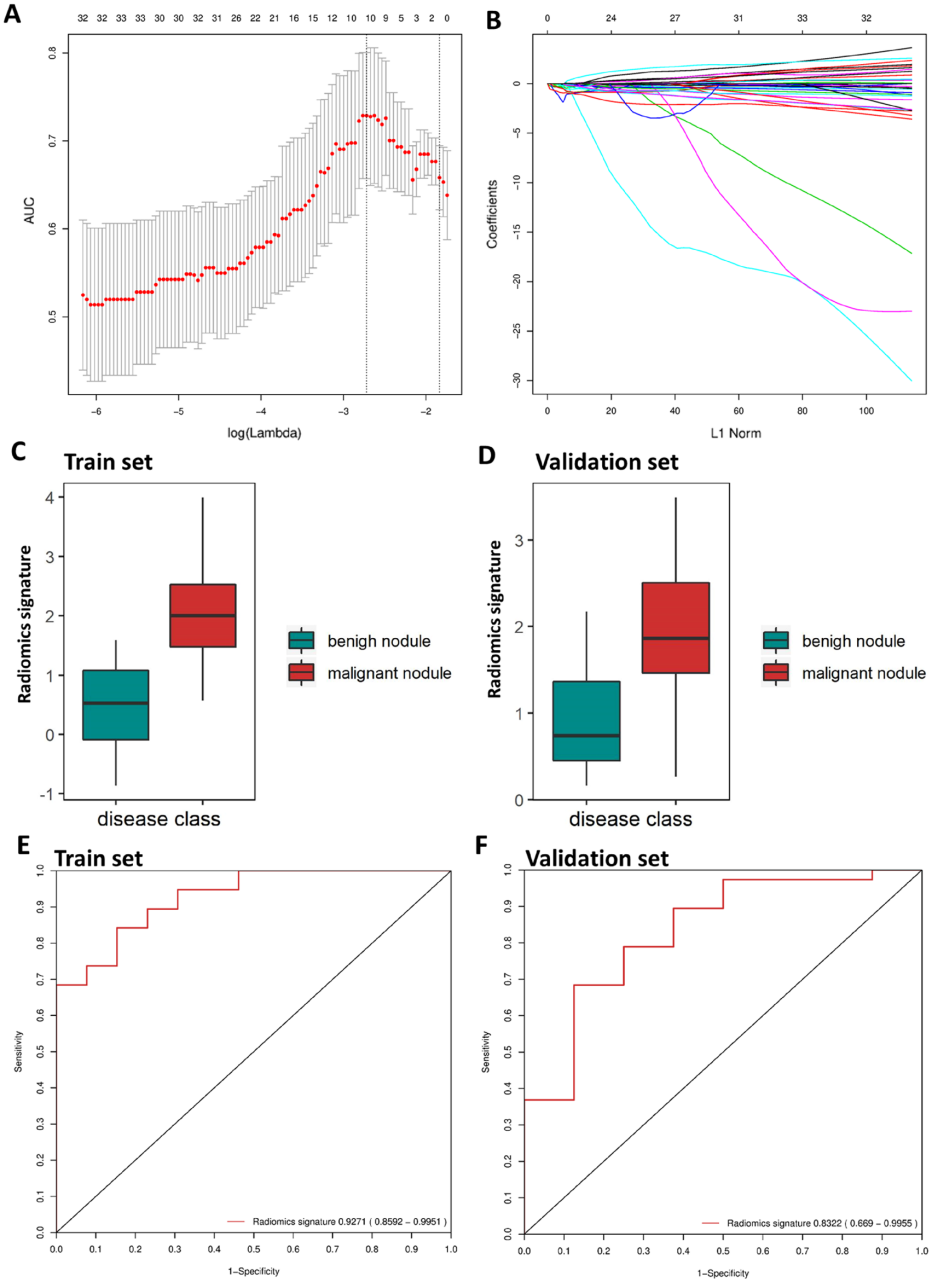
Texture feature selection using LASSO logistic regression and predictive accuracy of the radiomics signature. (**A**) Selection of the tuning parameter (λ) in the LASSO model via fivefold cross-validation based on maximum criteria. The predicted AUC from the LASSO regression cross-validation procedure was plotted as a function of log(λ). The y-axis indicates the predicted AUC. The lower x-axis indicates the log(λ). Numbers along the upper x-axis represent the average number of predictors. Red dots indicate the average predicted AUC for each model with a given λ, and vertical bars through the red dots show the upper and lower values of the predicted AUC. The vertical black lines define the optimal values of l, where the model provides its best fit to the data. An optimal λ value of 0.066 with log(λ) =  −2.72 was selected. (**B**) LASSO coefficient profiles of the 788 texture features. The dotted vertical line was plotted at the value selected using fivefold cross-validation in A. The ten resulting features with nonzero coefficients are indicated in the plot. Plots (**C**) and (**D**) present the boxplots of the radiomics score in the training and validation sets, respectively. Plots (**E**) and (**F**) show the receiver operating characteristic (ROC) curves of the radiomics signature in the training and validation sets, respectively.

Ten features of benign and malignant early-stage SPNs with nonzero coefficients were screened using the LASSO logistic regression model which was tuned using fivefold cross-validation on 70 patients in the training set (Fig. 2A,B). Nine of ten features demonstrated significant association with malignancy risk (Table S2). Among the 10 features we had included, only one original shape feature, and the other 9 were wavelet features. Therefore, the more complex features extracted after image transformation had stronger prediction and distinguishing ability, and were more suitable for identifying early-stage SPNs with LDCT. The radiomics score calculation formula and the selected features are presented in Supplementary Material S4. Malignant nodules generally displayed a higher radiomics score than benign nodules. There was a significant difference between the radiomics scores [median (interquartile range)] of the benign and malignant groups in the training set [0.525 (− 0.087 to 1.080) vs. 2.002 (1.475–2.523), respectively, *P* < 0.001]; this difference was confirmed in the validation set [0.739 (0.452–1.366) vs. 1.862 (1.462–2.507), respectively, *P* = 0.004] (Fig. 2C,D). The radiomics signature showed favorable predictive efficacy, with an AUC of 0.9393 [95% confidence interval (CI), 0.8799–0.9986] in the training set and 0.8257 (95% CI 0.6938–0.9576) in the validation set (Fig. 2E,F). In addition, an optimal radiomics score cutoff value of 1.64 was defined based on the maximum Youden index of all patients.

**Figure 3:**
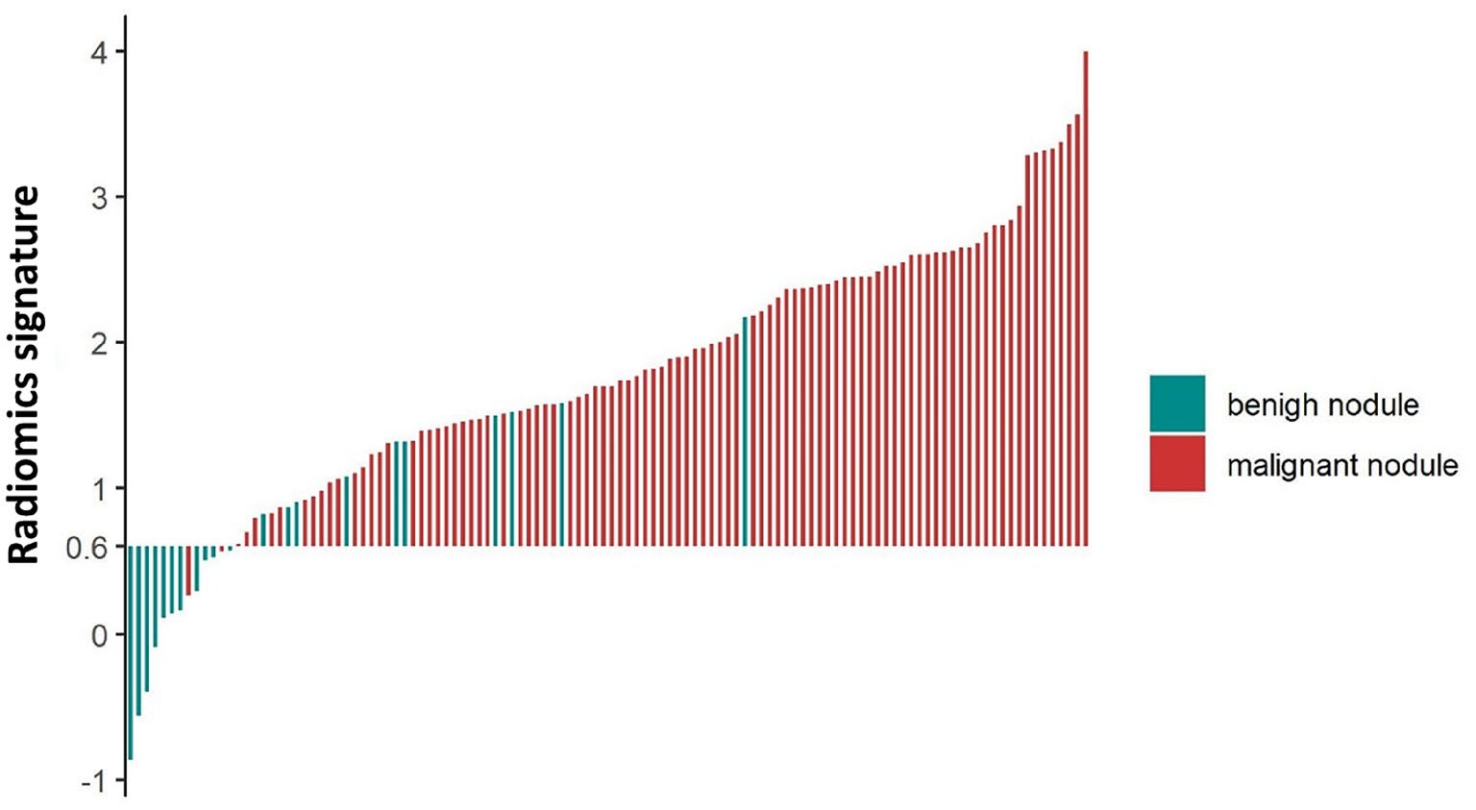
Waterfall plot for distribution of radiomics score and benign and malignant status of individual lesions. The radiomics score for each lesion in the study is shown here.

The radiomics signature was identified as an independent predictor of malignant early-stage SPNs in a multivariate logistic regression model (Table 2). The waterfall plot for the distribution of the radiomics score and benign and malignant status of individual lesions is presented in Fig. 3, which clearly reveals that almost all patients with malignant pulmonary nodules (97.9%, 93/95) would avoid being missed by using a cutoff value of the radiomics signature of 0.6.

**Table 2:** Risk factors for malignant in lung nodule.

Variable and intercept	Univariate logistic regression			Multivariate logistic regression		
	*β*	SE	*P*	*β*	SE	*P*
Radiomics signature	2.933	0.836	< 0.001	3.465	1.022	0.001
Age	0.053	0.031	0.087	0.123	0.068	0.069
Diameter	− 0.066	0.046	0.149	NA	NA	NA
Mayo score	0.118	0.223	0.597	NA	NA	NA
Gender	− 0.049	0.616	0.937	NA	NA	NA
Spicule sign	0.294	0.629	0.640	NA	NA	NA
Smoke	0.152	0.630	0.810	NA	NA	NA
Tumor history	16.161	1978.090	0.993	NA	NA	NA
Location	1.058	0.658	0.108	NA	NA	NA

### Construction, performance assessment and validation of the radiomics nomogram

**Figure 4:**
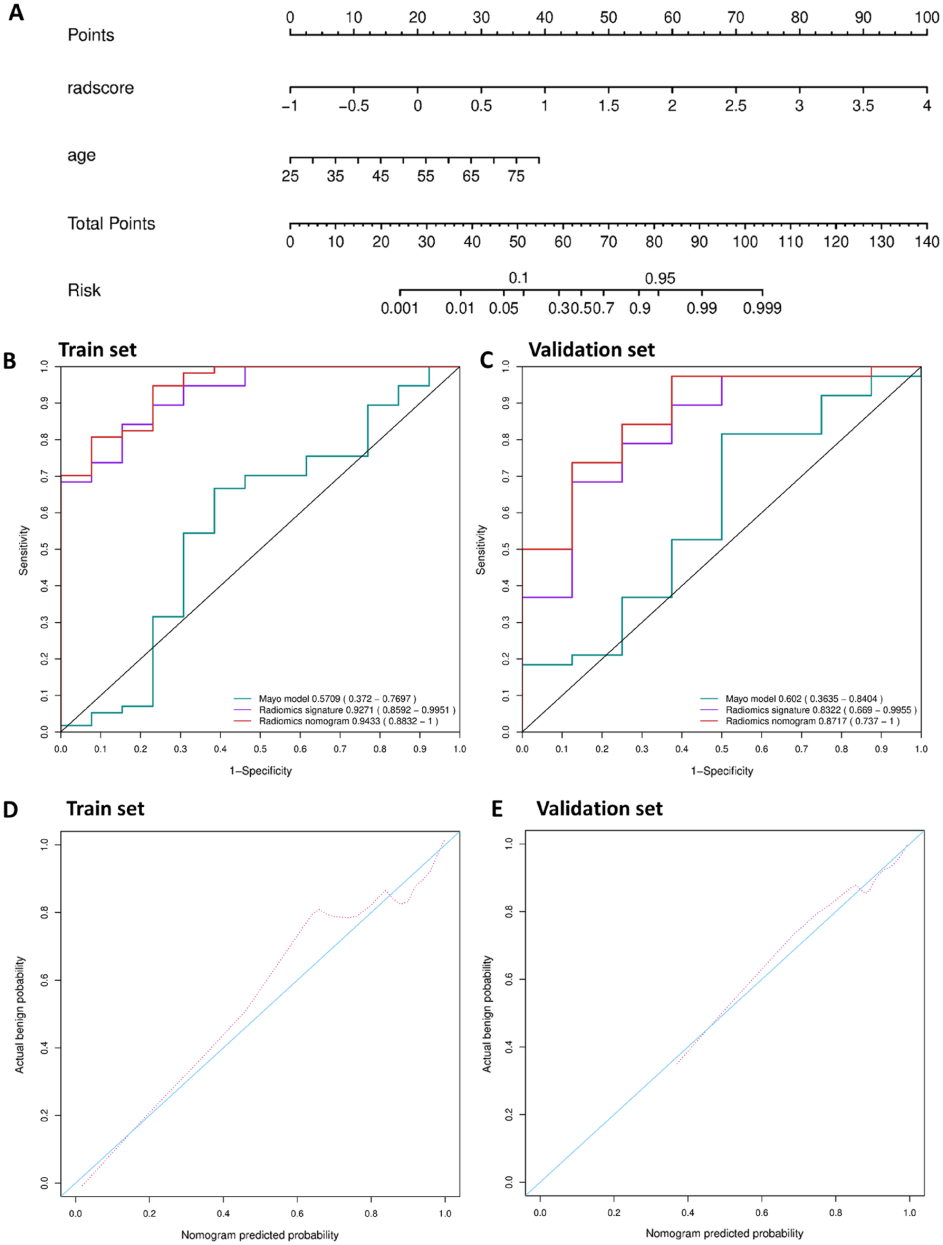
Radiomics nomogram for the prediction of benign and malignant early-stage SPNs. (**A**) A radiomics nomogram of the two predictors was constructed. (**B**) The AUC of 0.9433 (95% CI 0.8832–1) revealed good discrimination by the nomogram. (**C**) The AUC of the validation set was 0.8717 (95% CI 0.737–1). (**D**) The calibration curve and a nonsignificant Hosmer–Lemeshow test statistic (*P* = 0.9742) showed good calibration in the training set. (**E**) The Hosmer–Lemeshow test yielded a nonsignificant *P* value of 0.7410.

**Figure 5:**
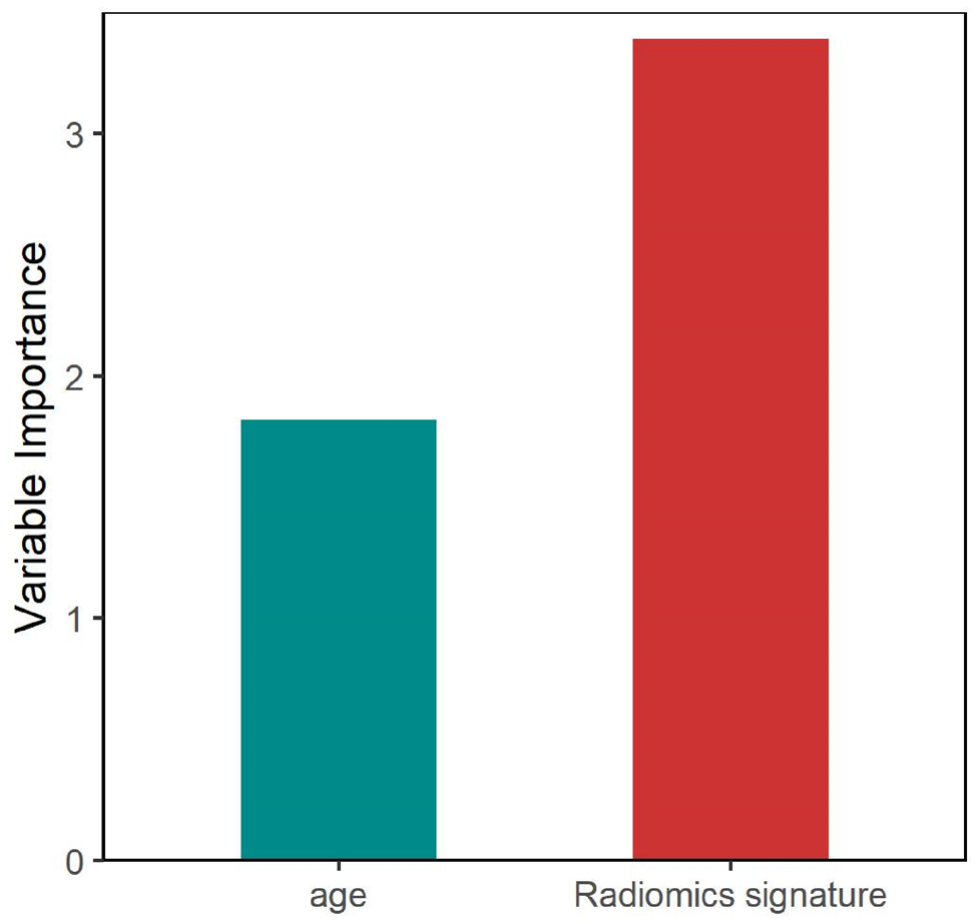
Variable importance of each variable in the radiomics nomogram.

A radiomics nomogram of the two predictors was constructed (Fig. 4A). The AUC of 0.9433 (95% CI 0.8832–1) revealed good discrimination by the nomogram (Fig. 4B). The calibration curve and a nonsignificant Hosmer–Lemeshow test statistic (*P* = 0.9742) showed good calibration in the training set (Fig. 4D). The AUC of the validation set was 0.8717 (95% CI 0.737–1; Fig. 4C), and the Hosmer–Lemeshow test yielded a nonsignificant *P* value of 0.7410 (Fig. 4E). Therefore, our nomogram performed well in both the training and validation sets. The radiomics signature presented relatively higher predictive importance than age in the radiomics nomogram (Fig. 5).

### Clinical usefulness of the radiomics nomogram

**Figure 6:**
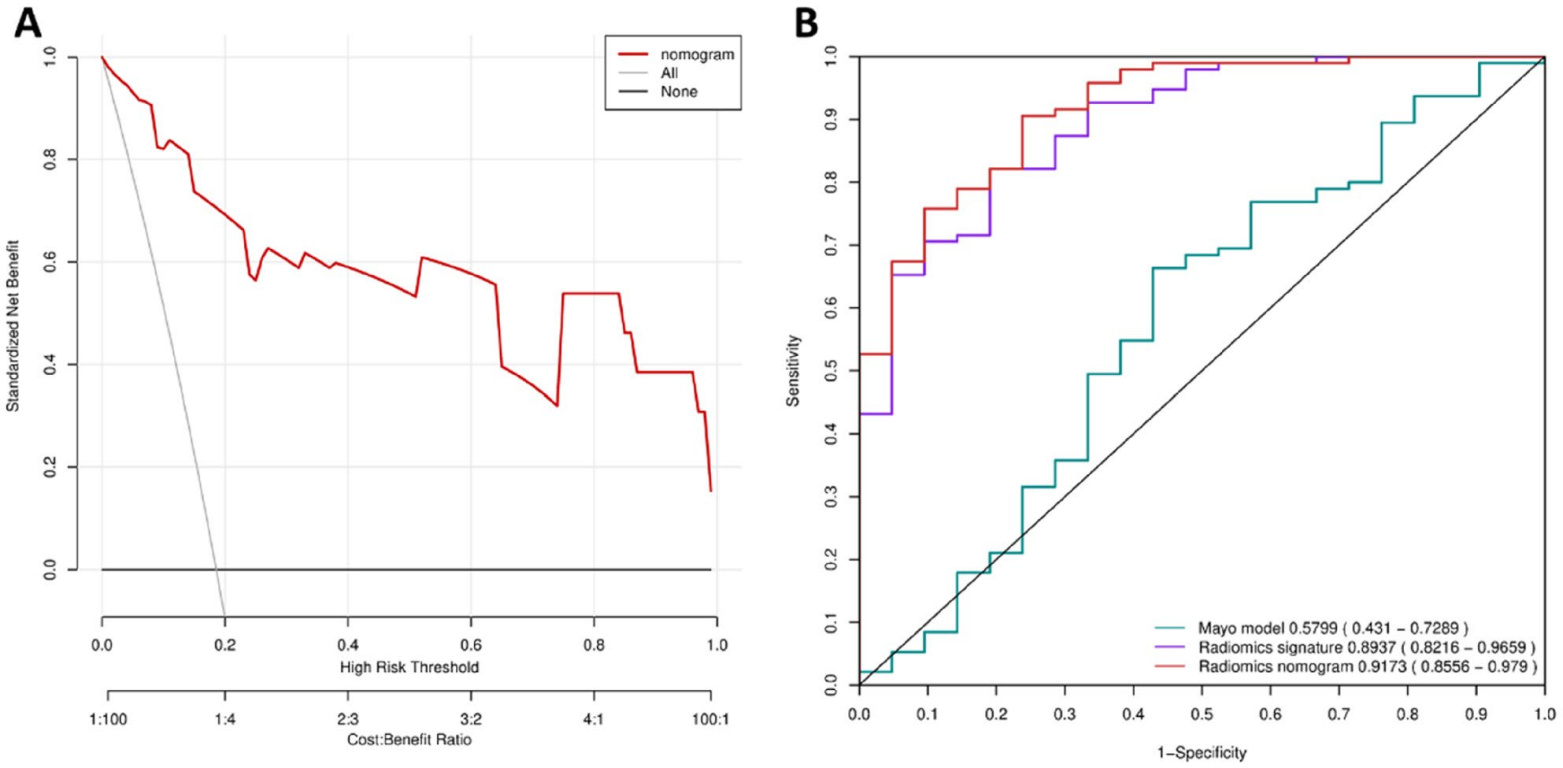
DCA for the radiomics nomogram and the ROC analyses of all 116 patients. (**A**) The y-axis represents the net benefit. The red line represents the radiomics nomogram. The gray line represents the hypothesis that all patients were malignant. The black line represents the hypothesis that no patients were malignant. The x-axis represents the threshold probability. The threshold probability is where the expected benefit of treatment is equal to the expected benefit of avoiding treatment. For example, if the possibility of malignant in a patient is over the threshold probability, then a treatment strategy for malignant should be adopted. The decision curves in the validation set showed that no matter what the threshold probability is, using the radiomics nomogram to predict malignant obtain more benefit than treating either all or no patients. (**B**) Performance of nomogram, radiomics signature and mayo model on all 116 patients. Nomogram adopt radiomics signature and age achieves best predict performance.

The DCA for the radiomics nomogram is presented in Fig. 6A. The DCA indicated that when the threshold probability for a doctor or a patient is within the range from 0 to 1, the radiomics nomogram adds more net benefit than the "treat all" or "treat none" strategies.

In addition, we evaluated the discriminatory efficiency of the radiomics nomogram in all 116 patients using ROC analyses. Figure 6B shows ROC analyses comparing the discriminatory efficacy of the radiomics nomogram to those of the radiomics signature and the patient age alone. The radiomics nomogram yielded the greatest ROC of 0.9173 (95% CI 0.8556–0.9790), which suggested that the nomogram achieved better predictive efficacy than either the radiomics signature or age alone.

## Discussion

Distinguishing benign and malignant pulmonary nodules and masses is critical in the diagnosis of lung diseases. Accurate prediction of benign and malignant lung lesions will allow appropriate clinical treatment and biopsy strategies. As the awareness of the importance medical technology and physical examination increases, more and more early-stage lesions are discovered. Among them, early-stage SPNs (≤ 3 cm) have few significant imaging features for diagnosis, preoperative noninvasive discrimination is difficult. Noninvasively distinguishing benign and malignant SPNs will provide considerable benefit for guiding clinical diagnosis and treatment. On the one hand, for benign lesions, choosing drug therapy or needle biopsy will significantly reduce the risk of surgery and avoid excessive medical treatment for patients. On the other hand, for malignant lesions, more active biopsy or surgical treatment will buy valuable time for the patient's recovery and maximize the benefits.

Lung cancer is one of the leading causes of cancer-related death worldwide and poses a serious threat to public health because most early lung cancer patients are asymptomatic, and symptoms only appear in the advanced stage^17^. Therefore, most lung cancer patients have distant metastasis at the time of initial diagnosis, resulting in a poor prognosis of lung cancer and a low survival rate^18^. The main difficulty and bottleneck at present is the lack of highly sensitive and specific diagnostic methods for early-stage lung cancer^19^. Lung cancer screening trials have shown that early detection can improve long-term survival in patients. Additionally, imaging examination plays an irreplaceable role in lung cancer detection, diagnosis and efficacy evaluation. With the development and improvement in CT, MRI, PET/CT, radiomics, and artificial intelligence technologies, diagnostic methods for lung cancer based on morphological, functional and molecular characteristics have been established^20^. More advanced imaging examinations, such as PET/CT, show better early diagnostic capabilities^21^; however, CT techniques, especially low-dose CT (LDCT), are simple, widespread, rapid and efficient and are a common means of early screening, diagnosis and evaluation of lung cancer. In addition, LDCT had a 24% positive rate for detecting lung nodules, and lung cancer-specific mortality was reduced by 20%. Therefore, early diagnosis is important for prognosis and survival. However, 96% of these 24% positive results were false positives^22^. Clinicians are still unable to correctly distinguish benign and malignant lesions based on preoperative imaging data, which seriously affects the accuracy of subsequent clinical decisions^23^. In addition to early imaging screening, lung cancer-specific tumor markers play an important role in early diagnosis and have been widely accepted by doctors and patients. Currently, carcinoembryonic antigen (CEA), neuron-specific enolase (NSE), cytokeratin 19 fragment (CYFRA21-1), pro-gastrin-releasing peptide (ProGRP) and squamous cell carcinoma (SCC) antigen are commonly used markers for the diagnosis of lung cancer^24^. However, the sensitivity and specificity of single tumor markers are low and can easily lead to a misdiagnosis. Although multi-index combined detection can improve the sensitivity and provide evidence for the early diagnosis of lung cancer, this method still requires further imaging confirmation.

Several predictive models (Mayo Clinic^25^, Veterans Association (VA)^26^, and Brock University^27^) using clinical and radiological features have been developed that can help physicians to distinguish between benign and malignant nodules^28^. These predictive models only included clinical values and radiological characteristics from CT, and there were no differences among the three models in determining the probability of malignancy of pulmonary nodules^29^. According to our study, the Mayo model had poor predictive ability for identifying early pulmonary nodules, probably because early pulmonary nodules have no obvious CT imaging features, such as spicule signs, which affected the efficacy of the model.

Radiomics is defined as the quantification of the phenotypic features of a lesion from medical imaging. Similarly, radiologists have already identified a relatively small number of qualitative visual physical characteristics to differentiate benign and malignant lesions and included in some predictive models such as the Mayo Clinic models^25^. The current challenge for radiomics is determining the most predictive features among thousands of potential phenotypic characteristics. Radiomics can be applied to lung cancer for the detection of lung cancer, prediction of malignancy, prediction of histology and subtype, prediction of prognosis, and assessment of treatment effect^30^. Hawkins et al. studied 600 patients with lung nodules graded from I to IV and only extracted 219 image features from LDCT^11^. Their radiomics classifier using random forests had an AUC of 0.87, which was equal to that of Paul’s convolutional neural network model^31^. Huang et al. included 186 lung nodules measuring less than 20 mm (a quarter were nonsolid lesions), extracted 1108 features, and used a random forest model that achieved an AUC of 0.91^32^. A support vector machine (SVM) model was used by Chen et al. in 72 patients, and only 4 features were selected to obtain an accuracy of 0.84^33^. Other models, such as the L1 regularized logistic regression model using only 94 radiomics features, obtained an AUC of 0.81 in the validation set without a reliable radiomics score formula^13^. In our study, we demonstrated that the LASSO logistic regression algorithm was a more effective model and that the second-order wavelet features were more suitable for identifying early-stage SPNs with LDCT. Among the 10 features we included, only one original shape feature, and the other 9 were wavelet features. By changing the ratio of high-frequency to low-frequency signal in images, we found that the wavelet transform increased the information of low-frequency signal, and extracted deeper and high-throughput features that is invisible to the naked eye. Other image transformations should also be considered in future research and would further improve our radiomics nomogram prediction capabilities. Our other advantage is that we did not distinguish between specific pathological types but developed a general recognition system for SPNs, which allows our model to be applied more extensively.

However, an important limitation that should be acknowledged in the current study is the relatively small sample size. With validation on more patients from multiple centers, it’s hopeful to improve the clinical applicability of the model in the current study. In addition, with the development of radiomics, more graphic transformation methods and radiomics features will be discovered and applied for the diagnosis and prediction of diseases. Correspondingly, more efficient and suitable machine learning and deep learning algorithms will be continuously applied to this field, and radiomics can be applied in more areas than just tumor research.

## Supplementary Information

Supplementary Information.

## Abbreviations

SPNs: Solitary pulmonary nodules
LDCT: Low-dose computed tomography
LASSO: The least absolute shrinkage and selection operator
AUC: Area under curve
CXR: Chest radiography
MRI: Magnetic resonance imaging
PET-CT: Positron emission tomography/computed tomography
CT: Computed tomography
MSCT: Multi-slice spiral computed tomography
HU: Hounsfield unit
PACS: Picture archiving and communication system
ROI: Regions of interest
ICCs: Interclass correlation coefficients
ROC: Receiver–operator characteristic
DCA: Decision curve analysis
SD: Standard deviation
CI: Confidence interval
NCCN: National comprehensive cancer network
CEA: Carcinoembryonic antigen
NSE: Neuron-specific enolase
CYFRA21-1: Cytokeratin 19 fragment
ProGRP: Pro-gastrin-releasing peptide
SCC: Squamous cell carcinoma
VA: Veterans association
SVM: Support vector machine
