# Supplementary material for: A wavelet features derived radiomics nomogram for prediction of malignant and benign early-stage lung nodules
Source: Sci Rep. 2021 Nov 16;11:22330. doi: 10.1038/s41598-021-01470-5 (PMC8595377; doi:10.1038/s41598-021-01470-5)
Supplement: Supplementary file 1 — Supplementary Information. [file 41598_2021_1470_MOESM1_ESM.docx]

**Supplementary Materials:**

Table S1. Baseline characteristics of the training and validation sets

| Characteristic | Training set (*N*=70) | | *P* | Validation set (*N*=46) | | *P* |
| --- | --- | --- | --- | --- | --- | --- |
|  | Malignant  (*N*=13) | Benign  (*N*=57) |  | Malignant  (*N*=8) | Benign  (*N*=38) |  |
| Age, mean±SD, years | 55.077 ± 9.438 | 62.246 ± 9.838 | 0.017 | 58.625 ± 14.530 | 62.947 ± 8.847 | 0.265 |
| Mayo score, mean±SD | -1.323 ± 1.281 | -0.954 ± 1.469 | 0.403 | -1.417 ± 2.146 | -1.012 ± 1.422 | 0.505 |
| SPN Diameter, mean±SD, mm | 18.077 ± 8.361 | 15.825 ± 6.285 | 0.274 | 16.000 ± 9.150 | 16.579 ± 6.425 | 0.830 |
| Gender |  |  | >0.999 |  |  | >0.999 |
| Male | 5 (38.462) | 24 (42.105) |  | 4 (50.000) | 21 (55.263) |  |
| Female | 8 (61.538) | 33 (57.895) |  | 4 (50.000) | 17 (44.737) |  |
| Spicule sign |  |  | 0.264 |  |  | >0.999 |
| 0 | 9 (69.231) | 27 (47.368) |  | 5 (62.500) | 22 (57.895) |  |
| 1 | 4 (30.769) | 30 (52.632) |  | 3 (37.500) | 16 (42.105) |  |
| Smoke |  |  | >0.999 |  |  | 0.661 |
| 0 | 7 (53.846) | 30 (52.632) |  | 4 (50.000) | 25 (65.789) |  |
| 1 | 6 (46.154) | 27 (47.368) |  | 4 (50.000) | 13 (34.211) |  |
| Tumor history |  |  | >0.999 |  |  | 0.787 |
| 0 | 13 (100.000) | 55 (96.491) |  | 8 (100.000) | 34 (89.474) |  |
| 1 | 0 (0.000) | 2 (3.509) |  | 0 (0.000) | 4 (10.526) |  |
| Location |  |  | 0.646 |  |  | 0.800 |
| Superior lobe | 6 (46.154) | 33 (57.895) |  | 3 (37.500) | 19 (50.000) |  |
| Other location | 7 (53.846) | 24 (42.105) |  | 5 (62.500) | 19 (50.000) |  |


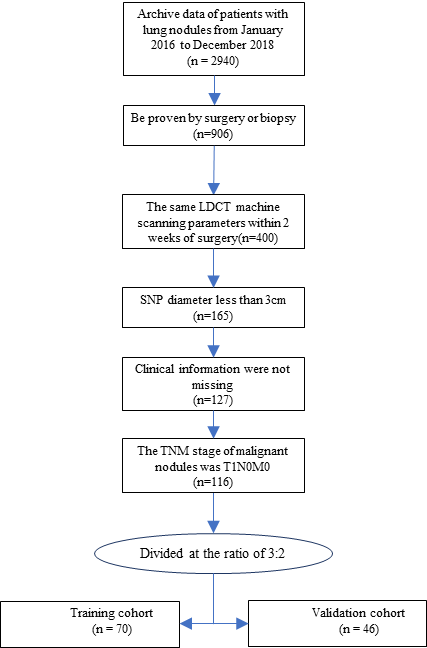


**Figure S1. Recruit pathway and the inclusion and exclusion criteria.**

The inclusion criteria were as follows: (1) Patients were proven lung lesion by surgical resection or image-guided biopsy pathology (n=906); (2) All patients underwent routine plain low-dose computed tomography (LDCT) of the entire thorax using the same CT machine with normalized reconstruction algorithm and thickness (1.5 mm), within two weeks of surgery (n=400); (3) CT was retrospectively analyzed to be solitary pulmonary nodules and diameter of the lesion was less than 3cm (n=165); (4) Clinical information were not missing (n=127); (5) The TNM stage of malignant nodules was T1N0M0 and was a primary tumor of the lung (n=116).

The exclusion criteria were as follows: (1) Patients with a history of other malignancies or other diseases affecting the lungs (exclude 2 from 165 SPN ≤3cm); (2) Patients whom under anti-tumor therapy before operation (exclude 0); (3) Patients in whom the CT imaging was reconstructed using different algorithms or thicknesses or if the reconstruction was performed on a different CT machine (exclude 301 from 906 patients by surgery or biopsy); (4) Those in whom the nodule segmentation failed to meet the standard leading to inaccurately extracted radiomics parameters (exclude 10 from 400 standard LDCT images).

**Supplementary Materials S2. Radiomic feature detailed information**

In this study, a total of 778 radiomics features were extracted, of which 100 features were extracted before wavelet decomposition and the remaining 688 were extracted after wavelet decomposition. All features were extracted using the PyRadiomics (<https://pyradiomics.readthedocs.io>). The 100 features were divided into three categories: 18 first order statistics features, 14 shape and size features and 68 textural features. The 678 features were classified as the fourth category, which contain all first-order statistics features and textural features but were extracted from images with wavelet decomposition.

**S2.1. First-order statistics features:**

First-order statistics features describe the gray level distribution of all voxels within the ROI^[1]^.

Let:

X be a set of all voxels include in the ROI, Np is the number of voxels in X.

P(i) be the first order histogram with Ng discrete intensity levels

p(i) be the normalized first order histogram and equal to P(i)/Np

c is optional value, which shifts the intensities to prevent negative values in X

1. Energy

$energy=\sum_{i=1}^{N_{p}} (\mathbf{X}(i)+c)^{2}$

1. Total energy

$$totalenergy=V_{voxel}\sum_{i=1}^{N_{p}} (\mathbf{X}(i)+c)^{2}$$

1. Entropy


 is an arbitrarily small positive number ($\approx2.2\times10^{-16}$)

1. Minimum

$$minimum=\min(\mathbf{X})$$

1. 10th percentile

The 10^th^ percentile of X

1. 90^th^ percentile

The 90^th^ percentile of X

1. Maximum

$$maximum=\max(\mathbf{X})$$

1. Mean

$$mean=\frac{1}{N_{p}}\sum_{i=1}^{N_{p}} \mathbf{X}(i)$$

1. Median

The median gray level intensity within the ROI.

1. Ineterquartile Range

$$interquartilerange=\mathbf{P}_{75}-\mathbf{P}_{25}$$

1. Range

$$range=\max(\mathbf{X})-\min(\mathbf{X})$$

1. Mean absolute deviation

$$MAD=\frac{1}{N_{p}}\sum_{i=1}^{N_{p}} |\mathbf{X}(i)-\bar{X}|$$

1. Robust Mean Absolute Deviation (rMAD)

$$rMAD=\frac{1}{N_{10-90}}\sum_{i=1}^{N_{10-90}} |\mathbf{X}_{10-90}(i)-\bar{X}_{10-90}|$$

1. Root Mean Squared (RMS)

$$RMS=\sqrt{\frac{1}{N_{p}}\sum_{i=1}^{N_{p}} (\mathbf{X}(i)+c)^{2}}$$

1. Skewness

$$skewness=\frac{\mu_{3}}{\sigma^{3}}=\frac{\frac{1}{N_{p}}\sum_{i=1}^{N_{p}} (\mathbf{X}(i)-\bar{X})^{3}}{\left(\sqrt{\frac{1}{N_{p}}\sum_{i=1}^{N_{p}} (\mathbf{X}(i)-\bar{X})^{2}}\right)^{3}}$$

1. Kurtosis

$$kurtosis=\frac{\mu_{4}}{\sigma^{4}}=\frac{\frac{1}{N_{p}}\sum_{i=1}^{N_{p}} (\mathbf{X}(i)-\bar{X})^{4}}{\left(\frac{1}{N_{p}}\sum_{i=1}^{N_{p}} (\mathbf{X}(i)-\bar{X})^{2}\right)^{2}}$$

1. Variance

$$variance=\frac{1}{N_{p}}\sum_{i=1}^{N_{p}} (\mathbf{X}(i)-\bar{X})^{2}$$

1. Uniformity

$$uniformity=\sum_{i=1}^{N_{g}} p(i)^{2}$$

**S2.2. Shape and size features:**

This group of features describe the three-dimensional size and shape of the ROI. Features were derived from a triangle mesh generated using a marching cubes algorithm based on the ROI^[]^.

Let:

Nv represent the number of voxels included in the ROI

Nf represent the number of faces defining the Mesh

V the volume of the mesh in mm3

A the suface area of the mesh in mm2

1. Mesh Volume

$$\begin{matrix} V_{i}=\frac{Oa_{i}\cdot(Ob_{i}\times Oc_{i})}{6}\text{ } \\ V=\sum_{i=1}^{N_{f}} V_{i}\text{ } \end{matrix}$$

1. Voxel Volume

$$V_{voxel}=\sum_{k=1}^{N_{v}} V_{k}$$

1. Surface Area

$$\begin{matrix} A_{i}=\frac{1}{2}|\text{a}_{i}\text{b}_{i}\times\text{a}_{i}\text{c}_{i}|\text{ } \\ A=\sum_{i=1}^{N_{f}} A_{i}\text{ } \end{matrix}$$

1. Surface Area to Volume ratio

$$surfacetovolumeratio=\frac{A}{V}$$

1. Sphericity

1. Maximum 3D diameter
2. Maximum 2D diameter (Slice)
3. Maximum 2D diameter (Column)
4. Maximum 2D diameter (Row)
5. Major Axis Length

$$majoraxis=4\sqrt{\lambda_{major}}$$

1. Minor Axis Length

$$minoraxis=4\sqrt{\lambda_{minor}}$$

1. Least Axis Length

$$leastaxis=4\sqrt{\lambda_{least}}$$

1. Elongation

$$elongation=\sqrt{\frac{\lambda_{minor}}{\lambda_{major}}}$$

1. Flatness

$$flatness=\sqrt{\frac{\lambda_{least}}{\lambda_{major}}}$$

**S2.3. Textural features:**

Textural features reflect information about the spatial arrangement of voxel intensities and therefore could describe the homogeneity of ROI. In our study, textural features were derived from four statistical feature matrices: 22 from the Gray-level co-occurrence matrix (GLCM), 16 from the Gray-level run-length texture matrix (GLRLM), 16 from the gray level size zone matrix (GLSZM) and 14 from the gray level dependence matrix (GLDM).

**S2.3.1 Gray-level co-occurrence matrix (GLCM)**

1. Autocorrelation

$$autocorrelation=\sum_{i=1}^{N_{g}} \sum_{j=1}^{N_{g}} p(i,j)ij$$

2. Joint Average

$$jointaverage=\mu_{x}=\sum_{i=1}^{N_{g}} \sum_{j=1}^{N_{g}} p(i,j)i$$

3. Cluster Prominence

$$clusterprominence=\sum_{i=1}^{N_{g}} \sum_{j=1}^{N_{g}} (i+j-\mu_{x}-\mu_{y})^{4}p(i,j)$$

4. Cluster Shade

$$clustershade=\sum_{i=1}^{N_{g}} \sum_{j=1}^{N_{g}} (i+j-\mu_{x}-\mu_{y})^{3}p(i,j)$$

5. Cluster Tendency

$$clustertendency=\sum_{i=1}^{N_{g}} \sum_{j=1}^{N_{g}} (i+j-\mu_{x}-\mu_{y})^{2}p(i,j)$$

6. Contrast

$$contrast=\sum_{i=1}^{N_{g}} \sum_{j=1}^{N_{g}} (i-j)^{2}p(i,j)$$

7. Correlation

$$correlation=\frac{\sum_{i=1}^{N_{g}} \sum_{j=1}^{N_{g}} p(i,j)ij-\mu_{x}\mu_{y}}{\sigma_{x}(i)\sigma_{y}(j)}$$

8. Difference Average

$$differenceaverage=\sum_{k=0}^{N_{g}-1} kp_{x-y}(k)$$

9. Difference Entropy

10. Difference Variance

$$differencevariance=\sum_{k=0}^{N_{g}-1} (k-DA)^{2}p_{x-y}(k)$$

11. Joint Energy

$$jointenergy=\sum_{i=1}^{N_{g}} \sum_{j=1}^{N_{g}} (p(i,j))^{2}$$

12. Joint Entropy

13. Informational Measure of Correlation (IMC) 1

$$IMC1=\frac{HXY-HXY1}{\max\{HX,HY\}}$$

14. Informational Measure of Correlation (IMC) 2

$$IMC2=\sqrt{1-e^{-2(HXY2-HXY)}}$$

15. Inverse Difference Moment (IDM)

$$IDM=\sum_{k=0}^{N_{g}-1} \frac{p_{x-y}(k)}{1+k^{2}}$$

16. Inverse Difference Moment Normalized (IDMN)

$$IDMN=\sum_{k=0}^{N_{g}-1} \frac{p_{x-y}(k)}{1+\left(\frac{k^{2}}{N_{g}^{2}}\right)}$$

17. Inverse Difference (ID)

$$ID=\sum_{k=0}^{N_{g}-1} \frac{p_{x-y}(k)}{1+k}$$

18. Inverse Difference Normalized (IDN)

$$IDN=\sum_{k=0}^{N_{g}-1} \frac{p_{x-y}(k)}{1+\left(\frac{k}{N_{g}}\right)}$$

19. Inverse Variance

$$inversevariance=\sum_{k=1}^{N_{g}-1} \frac{p_{x-y}(k)}{k^{2}}$$

20. Maximum Probability

$$maximumprobability=\max(p(i,j))$$

21. Sum Entropy

22. Sum of Squares

$$sumsquares=\sum_{i=1}^{N_{g}} \sum_{j=1}^{N_{g}} (i-\mu_{x})^{2}p(i,j)$$

**S2.3.2 Gray-level run-length texture matrix (GLRLM)**

1. Short Run Emphasis (SRE)

$$SRE=\frac{\sum_{i=1}^{N_{g}} \sum_{j=1}^{N_{r}} \frac{\mathbf{P}(i,j|\theta)}{j^{2}}}{N_{r}(\theta)}$$

2. Long Run Emphasis (LRE)

$$LRE=\frac{\sum_{i=1}^{N_{g}} \sum_{j=1}^{N_{r}} \mathbf{P}(i,j|\theta)j^{2}}{N_{r}(\theta)}$$

3. Gray Level Non-Uniformity (GLN)

$$GLN=\frac{\sum_{i=1}^{N_{g}} \left(\sum_{j=1}^{N_{r}} \mathbf{P}(i,j|\theta)\right)^{2}}{N_{r}(\theta)}$$

4. Gray Level Non-Uniformity Normalized (GLNN)

$$GLNN=\frac{\sum_{i=1}^{N_{g}} \left(\sum_{j=1}^{N_{r}} \mathbf{P}(i,j|\theta)\right)^{2}}{N_{r}(\theta)^{2}}$$

5. Run Length Non-Uniformity (RLN)

$$RLN=\frac{\sum_{j=1}^{N_{r}} \left(\sum_{i=1}^{N_{g}} \mathbf{P}(i,j|\theta)\right)^{2}}{N_{r}(\theta)}$$

6. Run Length Non-Uniformity Normalized (RLNN)

$$RLNN=\frac{\sum_{j=1}^{N_{r}} \left(\sum_{i=1}^{N_{g}} \mathbf{P}(i,j|\theta)\right)^{2}}{N_{r}(\theta)^{2}}$$

7. Run Percentage (RP)

$$RP=\frac{N_{r}(\theta)}{N_{p}}$$

8. Gray Level Variance (GLV)

$$GLV=\sum_{i=1}^{N_{g}} \sum_{j=1}^{N_{r}} p(i,j|\theta)(i-\mu)^{2}$$

9. Run Variance (RV)

$$RV=\sum_{i=1}^{N_{g}} \sum_{j=1}^{N_{r}} p(i,j|\theta)(j-\mu)^{2}$$

10. Run Entropy (RE)

11. Low Gray Level Run Emphasis (LGLRE)

$$LGLRE=\frac{\sum_{i=1}^{N_{g}} \sum_{j=1}^{N_{r}} \frac{\mathbf{P}(i,j|\theta)}{i^{2}}}{N_{r}(\theta)}$$

12. High Gray Level Run Emphasis (HGLRE)

$$HGLRE=\frac{\sum_{i=1}^{N_{g}} \sum_{j=1}^{N_{r}} \mathbf{P}(i,j|\theta)i^{2}}{N_{r}(\theta)}$$

13. Short Run Low Gray Level Emphasis (SRLGLE)

$$SRLGLE=\frac{\sum_{i=1}^{N_{g}} \sum_{j=1}^{N_{r}} \frac{\mathbf{P}(i,j|\theta)}{i^{2}j^{2}}}{N_{r}(\theta)}$$

14. Short Run High Gray Level Emphasis (SRHGLE)

$$SRHGLE=\frac{\sum_{i=1}^{N_{g}} \sum_{j=1}^{N_{r}} \frac{\mathbf{P}(i,j|\theta)i^{2}}{j^{2}}}{N_{r}(\theta)}$$

15. Long Run Low Gray Level Emphasis (LRLGLE)

$$LRLGLRE=\frac{\sum_{i=1}^{N_{g}} \sum_{j=1}^{N_{r}} \frac{\mathbf{P}(i,j|\theta)j^{2}}{i^{2}}}{N_{r}(\theta)}$$

16. Long Run High Gray Level Emphasis (LRHGLE)

$$LRHGLRE=\frac{\sum_{i=1}^{N_{g}} \sum_{j=1}^{N_{r}} \mathbf{P}(i,j|\theta)i^{2}j^{2}}{N_{r}(\theta)}$$

**S2.3.3 gray level size zone matrix (GLSZM)**

1. Small Area Emphasis (SAE)

$$SAE=\frac{\sum_{i=1}^{N_{g}} \sum_{j=1}^{N_{s}} \frac{\mathbf{P}(i,j)}{j^{2}}}{N_{z}}$$

2. Large Area Emphasis (LAE)

$$LAE=\frac{\sum_{i=1}^{N_{g}} \sum_{j=1}^{N_{s}} \mathbf{P}(i,j)j^{2}}{N_{z}}$$

3. Gray Level Non-Uniformity (GLN)

$$GLN=\frac{\sum_{i=1}^{N_{g}} \left(\sum_{j=1}^{N_{s}} \mathbf{P}(i,j)\right)^{2}}{N_{z}}$$

4. Gray Level Non-Uniformity Normalized (GLNN)

$$GLNN=\frac{\sum_{i=1}^{N_{g}} \left(\sum_{j=1}^{N_{s}} \mathbf{P}(i,j)\right)^{2}}{N_{z}^{2}}$$

5. Size-Zone Non-Uniformity (SZN)

$$SZN=\frac{\sum_{j=1}^{N_{s}} \left(\sum_{i=1}^{N_{g}} \mathbf{P}(i,j)\right)^{2}}{N_{z}}$$

6. Size-Zone Non-Uniformity Normalized (SZNN)

$$SZNN=\frac{\sum_{j=1}^{N_{s}} \left(\sum_{i=1}^{N_{g}} \mathbf{P}(i,j)\right)^{2}}{N_{z}^{2}}$$

7. Zone Percentage (ZP)

$$ZP=\frac{N_{z}}{N_{p}}$$

8. Gray Level Variance (GLV)

$$GLV=\sum_{i=1}^{N_{g}} \sum_{j=1}^{N_{s}} p(i,j)(i-\mu)^{2}$$

9. Zone Variance (ZV)

$$ZV=\sum_{i=1}^{N_{g}} \sum_{j=1}^{N_{s}} p(i,j)(j-\mu)^{2}$$

10. Zone Entropy (ZE)

11. Low Gray Level Zone Emphasis (LGLZE)

$$LGLZE=\frac{\sum_{i=1}^{N_{g}} \sum_{j=1}^{N_{s}} \frac{\mathbf{P}(i,j)}{i^{2}}}{N_{z}}$$

12. High Gray Level Zone Emphasis (HGLZE)

$$HGLZE=\frac{\sum_{i=1}^{N_{g}} \sum_{j=1}^{N_{s}} \mathbf{P}(i,j)i^{2}}{N_{z}}$$

13. Small Area Low Gray Level Emphasis (SALGLE)

$$SALGLE=\frac{\sum_{i=1}^{N_{g}} \sum_{j=1}^{N_{s}} \frac{\mathbf{P}(i,j)}{i^{2}j^{2}}}{N_{z}}$$

14. Small Area High Gray Level Emphasis (SAHGLE)

$$SAHGLE=\frac{\sum_{i=1}^{N_{g}} \sum_{j=1}^{N_{s}} \frac{\mathbf{P}(i,j)i^{2}}{j^{2}}}{N_{z}}$$

15. Large Area Low Gray Level Emphasis (LALGLE)

$$LALGLE=\frac{\sum_{i=1}^{N_{g}} \sum_{j=1}^{N_{s}} \frac{\mathbf{P}(i,j)j^{2}}{i^{2}}}{N_{z}}$$

16. Large Area High Gray Level Emphasis (LAHGLE)

$$LAHGLE=\frac{\sum_{i=1}^{N_{g}} \sum_{j=1}^{N_{s}} \mathbf{P}(i,j)i^{2}j^{2}}{N_{z}}$$

**S2.3.4 gray level dependence matrix (GLDM)**

1. Small Dependence Emphasis (SDE)

$$SDE=\frac{\sum_{i=1}^{N_{g}} \sum_{j=1}^{N_{d}} \frac{\mathbf{P}(i,j)}{i^{2}}}{N_{z}}$$

2. Large Dependence Emphasis (LDE)

$$LDE=\frac{\sum_{i=1}^{N_{g}} \sum_{j=1}^{N_{d}} \mathbf{P}(i,j)j^{2}}{N_{z}}$$

3. Gray Level Non-Uniformity (GLN)

$$GLN=\frac{\sum_{i=1}^{N_{g}} \left(\sum_{j=1}^{N_{d}} \mathbf{P}(i,j)\right)^{2}}{N_{z}}$$

4. Dependence Non-Uniformity (DN)

$$DN=\frac{\sum_{j=1}^{N_{d}} \left(\sum_{i=1}^{N_{g}} \mathbf{P}(i,j)\right)^{2}}{N_{z}}$$

5. Dependence Non-Uniformity Normalized (DNN)

$$DNN=\frac{\sum_{j=1}^{N_{d}} \left(\sum_{i=1}^{N_{g}} \mathbf{P}(i,j)\right)^{2}}{N_{z}^{2}}$$

6. Gray Level Variance (GLV)

$$GLV=\sum_{i=1}^{N_{g}} \sum_{j=1}^{N_{d}} p(i,j)(i-\mu)^{2}\text{, where}\mu=\sum_{i=1}^{N_{g}} \sum_{j=1}^{N_{d}} ip(i,j)$$

7. Dependence Variance (DV)

$$DV=\sum_{i=1}^{N_{g}} \sum_{j=1}^{N_{d}} p(i,j)(j-\mu)^{2}\text{, where}\mu=\sum_{i=1}^{N_{g}} \sum_{j=1}^{N_{d}} jp(i,j)$$

8. Dependence Entropy (DE)

9. Low Gray Level Emphasis (LGLE)

$$LGLE=\frac{\sum_{i=1}^{N_{g}} \sum_{j=1}^{N_{d}} \frac{\mathbf{P}(i,j)}{i^{2}}}{N_{z}}$$

10. High Gray Level Emphasis (HGLE)

$$HGLE=\frac{\sum_{i=1}^{N_{g}} \sum_{j=1}^{N_{d}} \mathbf{P}(i,j)i^{2}}{N_{z}}$$

11. Small Dependence Low Gray Level Emphasis (SDLGLE)

$$SDLGLE=\frac{\sum_{i=1}^{N_{g}} \sum_{j=1}^{N_{d}} \frac{\mathbf{P}(i,j)}{i^{2}j^{2}}}{N_{z}}$$

12. Small Dependence High Gray Level Emphasis (SDHGLE)

Measures the joint distribution of small dependence with higher gray-level values.

13. Large Dependence Low Gray Level Emphasis (LDLGLE)

$$LDLGLE=\frac{\sum_{i=1}^{N_{g}} \sum_{j=1}^{N_{d}} \frac{\mathbf{P}(i,j)j^{2}}{i^{2}}}{N_{z}}$$

14. Large Dependence High Gray Level Emphasis (LDHGLE)

$$LDHGLE=\frac{\sum_{i=1}^{N_{g}} \sum_{j=1}^{N_{d}} \mathbf{P}(i,j)i^{2}j^{2}}{N_{z}}$$

**References for S2**

[1] Lorensen WE, Cline HE. Marching cubes: A high resolution 3D surface construction algorithm. SIGGRAPH Comput. Graph.. 1987. 21(4): 163-169.


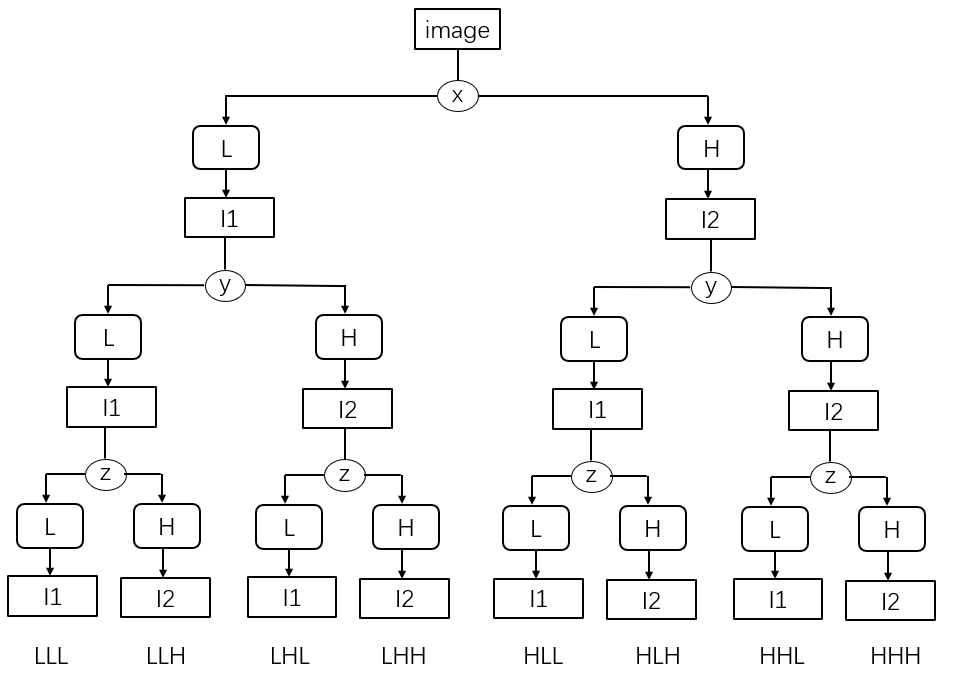


**Figure S3** **Diagram of a wavelet filter applied to a single image.** After wavelet decomposition, low-level information hidden from the human’s eye could be exposed. In our study, coiflet1 wavelet^[1]^ was used to decompose the original image. High and low pass filters were applied step on both x, y, z coordinates, which totally generate eight decompositions from one patient’s image. First-order statistics and textural features described above were extracted from all of eight decompositions. A total of 688 wavelet features were extracted.

**References for figure S3**

[1] Beylkin G, Coifman R, Rokhlin V. Fast wavelet transforms and numerical algorithms I. Commun Pure Appl Math. 1991. 44(2): 141-183.

**Supplementary Materials S4. Radiomics score calculation formula：**

Radiomics score= 3.8255- 0.2886×Original_firstorder_Maximum

- 0.3206×Wavelet_LLH_glcm_Difference Variance

-1.1648×Wavelet_LLH_glcm_Imc2

- 0.0403×Wavelet_LHL_gldm_Large Dependence High Gray Level Emphasis

-0.1214×Wavelet_LHH_glszm_Large Area High Gray Level Emphasis

-0.8135×Wavelet_HLL_glcm_Correlation

-0.0357×Wavelet_HLH_firstorder_Mean

-0.3794×Wavelet_HHH_glcm_Correlation

-0.1132×Wavelet_LLL_firstorder_Maximum

-0.0320×Wavelet_LLL_glcm_Difference Variance

Table S2. Association between radiomics features and malignancy risk in lung nodule

| Variables | Univariate logistic regression | | |
| --- | --- | --- | --- |
|  | *β* | SE | *P* |
| original_firstorder_Maximum | -0.997 | 0.322 | 0.002 |
| wavelet.LLH_glcm_DifferenceVariance | -1.479 | 0.587 | 0.012 |
| wavelet.LLH_glcm_Imc2 | -6.013 | 2.293 | 0.009 |
| wavelet.LHL_gldm_LargeDependenceHighGrayLevelEmphasis | -0.925 | 0.375 | 0.014 |
| wavelet.LHH_glszm_LargeAreaHighGrayLevelEmphasis | -0.558 | 0.274 | 0.042 |
| wavelet.HLL_glcm_Correlation | -4.410 | 1.444 | 0.002 |
| wavelet.HLH_firstorder_Mean | -0.607 | 0.367 | 0.098 |
| wavelet.HHH_glcm_Correlation | -1.709 | 0.627 | 0.006 |
| wavelet.LLL_firstorder_Maximum | -1.219 | 0.394 | 0.002 |
| wavelet.LLL_glcm_DifferenceVariance | -2.270 | 0.835 | 0.007 |
